# Separation of Teaching and Licensing

**Published:** 1851-09

**Authors:** 


*Separation of teaching and licensing.* — Our respected contemporary, the New York Med. Gazette, announces, as a novelty, the examination of candidates for graduation, at the New York Med. College, before censors who are in no way connected with the college. This plan has always been pursued at the University of Buffalo, and, also, at Geneva Med. College. A board of curators are connected with these institutions for the express purpose of attending examinations, and satisfying themselves of the fitness of candidates for graduation. The written consent of a certain number of curators, is a requisite for graduation provided by the charter of the University of Buffalo.

